# The Role of Vision in the Emergence of Mate Preferences

**DOI:** 10.1007/s10508-020-01901-w

**Published:** 2021-04-13

**Authors:** Meike Scheller, Francine Matorres, Anthony C. Little, Lucy Tompkins, Alexandra A. de Sousa

**Affiliations:** 1 Department of Psychology, University of Bath, Bath, UK; 2 School of Psychology, University of Aberdeen, Aberdeen, AB24 3FX UK; 3 Centre for Health and Cognition, Bath Spa University, Bath, UK

**Keywords:** Blindness, Visual impairment, Mate preferences, Sex differences, Partner choice, Sensory cues

## Abstract

Cross-cultural research has repeatedly demonstrated sex differences in the importance of partner characteristics when choosing a mate. Men typically report higher preferences for younger, more physically attractive women, while women typically place more importance on a partner’s status and wealth. As the assessment of such partner characteristics often relies on visual cues, this raises the question whether visual experience is necessary for sex-specific mate preferences to develop. To shed more light onto the emergence of sex differences in mate choice, the current study assessed how preferences for attractiveness, resources, and personality factors differ between sighted and blind individuals using an online questionnaire. We further investigate the role of social factors and sensory cue selection in these sex differences. Our sample consisted of 94 sighted and blind participants with different ages of blindness onset: 19 blind/28 sighted males and 19 blind/28 sighted females. Results replicated well-documented findings in the sighted, with men placing more importance on physical attractiveness and women placing more importance on status and resources. However, while physical attractiveness was less important to blind men, blind women considered physical attractiveness as important as sighted women. The importance of a high status and likeable personality was not influenced by sightedness. Blind individuals considered auditory cues more important than visual cues, while sighted males showed the opposite pattern. Further, relationship status and indirect, social influences were related to preferences. Overall, our findings shed light on the availability of visual information for the emergence of sex differences in mate preference.

**Supplementary Information:**

The online version contains supplementary material available at10.1007/s10508-020-01901-w.

## Introduction

The formation of close, romantic relationships plays a substantial role in individual development and is an integral part of human societies (Collins, Welsh, & Furman, 2008; Lindholm, 2006). The reasons for preferring and choosing a certain person over others to engage in a romantic relationship have received great interest from social and evolutionary scientists for several decades (Bech-Sørensen & Pollet, 2016; Buss, 1985; Hudson & Henze, 2006; Symons, 1979). A large body of research has demonstrated consistent patterns of sex differences but also commonalities in partner preferences of men and women (Buss, 2006; Buss, Shackelford, Kirkpatrick, & Larsen, 2001; Marzoli, Havlíček, & Roberts, 2018).

Partner preferences are the emotional, physical, and resource-related characteristics people prefer their partner to have (Buss & Barnes, 1986) and are used to evaluate potential partners in order to assess if they are more desirable than others. Commonalities between the sexes have been reported, highlighting that both men and women prefer attributes like reliability, emotional stability as well as mutual love and attraction (Buss et al., 2001). Differences, however, have been repeatedly demonstrated for the desirability of specific partner features (e.g., height, salary), which are usually grouped into attributes such as physical attractiveness or the ability to acquire resources and a high socioeconomic status. While men most commonly prefer a partner that displays youthfulness, physical attractiveness, and a desire for a home and children, women tend to consider a high status and the ability to provide good financial prospects as more important for men (Buss et al., 2001; Symons, 1979).

The directionality of these preferences has been shown to be consistent across time and cultures, having been replicated in more than 37 different countries over a long period of time (Buss, 1989; Sprecher, Sullivan, & Hatfield, 1994). Cross-cultural replications of the effect suggest that sex differences in mate preferences are evolutionarily adaptive and could indicate that there are genetic mechanisms at play, independent of individual experience. However, recent research also suggests that the magnitude of these sex differences has been decreasing over the past few decades, with male preferences for status and resources in a mate and female preferences for a physically attractive mate are both increasing (Bech-Sørensen & Pollet, 2016; Moore, Cassidy, & Perrett, 2010; Shackelford, Schmitt, & Buss, 2005; Zentner & Mitura, 2012). These findings have led to further investigations into the mechanisms underlying sex differences in mate preference and whether these are linked to evolutionarily adaptive mechanisms under genetic constraint or whether they are more flexible and responsive to changes in societal structure and the socialization of gender roles (Kavaliers, Matta, & Choleris, 2017; Zentner & Mitura, 2012).

Several theories have been put forward to explain why these sex differences occur; however, most of the ideas that have been proposed to date cannot disentangle which factors drive the emergence and persistence of sex-specific partner preferences. One way to allow for a better dissociation of these factors is to examine whether sex differences in mate preference depend on the availability of visual information. As vision plays an important role in the assessment of observable partner characteristics such as physical attractiveness, investigating the effects of blindness on mate preference allows us to gain more insight into the emergence and plasticity of sex differences in partner preference. Indeed, in a classic discussion of the evolution of sex differences in mate preferences, Symons (1979) noted that visual cues related to fertility (e.g., youth) would be more important to men than women because of differences in reproduction and investment and posited the question of how blind men judge attractiveness.

Thus far, however, only one study has quantitatively investigated sex differences in the physical and nonphysical aspects of mate preferences between sighted and blind individuals (Hasenkamp, Kümmerling, & Hassebrauck, 2005), in stark contrast to decades worth of research into this topic in the sighted. In their study, Hasenkamp et al. examined the importance of physical attractiveness and resource acquisition in potential romantic partners in congenitally blind and sighted individuals. Results showed that physical attractiveness and resource acquisition were valued less by blind men in comparison to sighted men. Blind women, on the other hand, exhibited a stronger preference for physical attractiveness than blind men. The importance of status and resource acquisition was overall lower for congenitally blind than for sighted individuals, independent of their sex. These results led Hasenkamp et al. to conclude that partner preferences were adapted to the individual’s own perceived “market value” (p. 81) although it is possible that devaluing physical attractiveness could be a direct consequence of the lack of visual experience of this trait.

Similar differences in preferences have also been reported for adolescents with visual impairments, who value physical attractiveness and material resources less than sighted individuals (Pinquart & Pfeiffer, 2012). This indicates how the availability and use of vision for assessing a potential partner’s qualities shapes our conception and preferences for an ideal partner. Pinquart and Pfeiffer further found that visually impaired individuals placed more importance on emotional maturity than sighted individuals, indicating further compensatory changes toward placing higher importance on features that are not necessarily visually assessed. By comparing visually impaired adolescents with residual vision to those that were totally blind, Pinquart and Pfeiffer also showed that physical attractiveness was considered even less important when vision was completely absent.

As blind individuals cannot visually assess the physical attractiveness of their partner, the value of visually perceivable attractiveness would be expected to be diminished in blind individuals. This diminished value would then be reflected in reduced importance ratings of their partner’s physical attractiveness. However, it is important to note that information about a potential partner can generally be obtained through multiple senses (e.g., vision, audition, olfaction, or touch). So while blind people might not be able to access visual partner features, physical attractiveness can still be perceived through the other senses. For example, a person’s height or weight can be judged from auditory cues, facial and bodily physical characteristics can be felt through touch, and hygiene and body odor composition can be smelled even without direct physical contact.

Notably, while physical traits could indeed be assessed through touch, it remains a misconception among many sighted people that touching another’s face or body is a common way for blind people to determine whether they are physically attractive. In personal communications, blind participants have reported to not explore a potential partner’s face or body haptically, unless a certain degree of intimacy is already established. This is consolidated by recent findings from Sorokowska, Oleszkiewicz, and Sorokowski (2018), who assessed differences between sighted and non-sighted individuals in sensory cue importance for mate selection. In their study, Sorokowska et al. asked blind and sighted participants to rate the importance of smell, touch, and audition for assessing partner characteristics. Their findings showed that auditory cues were considered more important in blind compared to sighted individuals. Touch, on the other hand, was considered the least important sense among blind individuals. The study further reported that olfactory cues were considered more important than other cues, especially in women assessing potential male partners (Sorokowska et al., 2018).

Indeed, in sighted individuals, sex differences for the importance of different sensory cues have already been reported previously. Two studies, conducted in the USA and the Czech Republic, showed that men considered vision the most important sense for mate choice, whereas women rated olfaction as the most important sense (Havlíček et al., 2008; Herz & Inzlicht, 2002). The heightened importance of odor for women in particular might be ascribed to the role odor plays in determining genetic quality and compatibility (Havlíček & Roberts, 2009). Yet, women also place greater importance on olfactory cues in social contexts that are not related to mate choice, potentially relating to offspring identification and food acquisition (Havlíček et al., 2008). Therefore, it remains unclear whether mate choice is driving the aforementioned sex differences.

Whether blind people consider visual cues important, independently of their direct accessibility, remains elusive. While visual cues, such as good looks, might not be directly perceived by an individual who is blind, they might bear an indirect advantage. A blind person could rate specific visual traits important in a partner if these traits are generally rated as more desirable within a society. Obtaining a more desirable mate may indirectly increase the individual’s own mating value.

So far, research on partner preferences of blind and visually impaired individuals is limited and explanations for any sex-specific deviations from partner preferences in the sighted are scarce. One factor that might allow us to better understand sex differences in partner preferences, both in the sighted and blind, is the individual’s perception that their partner’s appearance influences how they themselves are perceived. We will call these “indirect appearance effects” from here on. Indirect appearance effects could explain why having a physically attractive partner of high social status is valued beyond a person’s own preference. For example, previous studies suggest that having a physically attractive partner may enhance a person’s attractiveness to others. Little, Caldwell, Jones, and DeBruine (2011) found that a person’s own level of attractiveness can be increased when paired with an attractive partner model. The effect appeared to be more prominent for less attractive faces, indicating that having an attractive partner may be more beneficial for less attractive people (Little, Caldwell, Jones, & DeBruine, 2015). Furthermore, Winegard, Winegard, and Geary (2013) reported that both men and women flaunt attractive partners or conceal unattractive partners to same-age peers in order to increase their own desirability. Therefore, one could expect that the importance of physical attractiveness would be higher in individuals that believe that their partner’s appearance influences how others perceive them.

Another potential factor that might influence importance ratings of a partner’s physical attractiveness is the person’s relationship status. Individuals in relationships, in comparison to those who are single, have previously reported reduced importance of attractiveness ratings for the opposite sex (Karremans, Dotsch, & Corneille, 2011). Ritter and Karremans (2010) also showed that partnered participants were less interested in physically attractive individuals than participants that reported to be single. These findings have been explained as a means of maintaining one’s current relationship by preventing the pursuit of alternate partners.

The present study was conducted to better understand the role that vision plays for the development of sex differences in partner preferences. To his end, we assessed sex-specific partner preferences in blind and sighted individuals, employing methods that have been used previously by Hasenkamp et al. (2005). We predicted to replicate a reduction in the importance of status and resources in blind compared to sighted individuals, as well as a higher importance in women than men. Furthermore, we predicted that the importance of a partner’s physical attractiveness is reduced in blind men, but not in blind women.

The present study further expands on their findings by exploring potential reasons for the reported reversal of sex differences in the importance of physical attractiveness in blind individuals compared to sighted individuals. To do so, we assessed the indirect appearance effects, as well as the role of relationship status for the emergence of sex differences in both sighted and blind individuals. Based on previous research, we predicted that the importance of physical attractiveness would be higher in individuals that believe that their partner’s appearance influences how others perceive them. We further predicted similar effects for the importance of a high status and resource acquisition abilities, as these are partner characteristics that would allow an individual to indirectly increase their own value through a more desirable partner. According to findings from Ritter and Karremans (2010), as well as Karremans et al. (2011), we also predicted that partnered participants will place less importance on physical attractiveness in comparison to single participants.

Lastly, this study investigated how the importance of sensory cues differ between sexes in both blind and sighted men and women. We predicted that blind participants place more importance on non-visual traits such as a pleasant voice or good body odor, and women to place overall more importance on a pleasant odor.

## Method

### Participants

The sample consisted of 103 participants, 49 males (19 blind, 28 sighted, and 2 moderately visually impaired) and 54 females (19 blind, 28 sighted, and 7 moderately visually impaired). The sighted group included participants that were either fully sighted (*n* = 28) or had their vision corrected by lenses (*n* = 29). The blind group included participants who were registered blind or legally blind (*n* = 38). Participants who responded that they had low vision or were partially sighted (*n* = 9) were excluded from the data analysis as it was decided the sample was too small to compare with the blind and sighted groups. In the blind group, glaucoma (21%), retinitis pigmentosa (21%), and optic neuropathy (16%) were the most common reported causes of blindness. The age of onset for visual deprivation ranged from birth to 60 years old, with 53% of blind participants reporting to be blind since birth or developing blindness within the first 6 months of life. As all blind individuals were equally restricted in the direct access of visual characteristics of a potential partner, independently of the age of blindness onset, they were treated as one group. However, to confirm that grouping did not influence the main effects, importance ratings were assessed separately for congenitally blind and late blind individuals in Supplementary Material S2.

The age of sighted males ranged from 19 to 69 years (*M* = 35.4, *SD* = 16.12), whereas blind males ranged from 21 to 65 years (*M* = 38, *SD* = 16.41). For sighted females, age ranged from 16 to 55 years (*M* = 31.75, *SD* = 13.11), and for blind females from 18 to 68 years (*M *= 39.8, *SD* = 15.03). There was no significant age difference between sighted and blind participants (*t*[76] = 1.53, *p* = .130), and there was no significant age difference between males and females (*t*[91] = .57, *p* = .571). A two-factorial ANOVA showed that there was no significant interaction between sex and sightedness for age (*F*[1, 90] < 1, *p *= .481).

Sighted participants were recruited through opportunity sampling from Bath Spa University, University of Bath, and online advertisements. To obtain responses from blind participants, several charities and organizations for the blind were contacted via email and social media. Ethical approval was obtained from the Bath Spa University Psychology Ethics Board.

### Materials

Preference scores for different potential partner characteristics were collected using a standardized questionnaire survey on mate preferences, adapted from Hasenkamp et al. (2005) (see Supplementary Material S3). The list of assessed preferential partner traits included in the questionnaire was predominantly based on the mate selection surveys of Hudson and Henze (2006) and Christensen (1947). These partner traits have been reported to have cross-cultural reliability as examined by Buss (1989), who assessed their robustness across 37 different cultures. These questions were mainly used to investigate the roles of partner traits in mate preferences. The questionnaire contained questions about the importance of partner traits that were also used to assess the importance of good looks, a pleasant voice, and a pleasant odor. In addition, the questionnaire also included questions asking participants about their relationship status and about whether they believed that their partner’s appearance influenced how others perceived them.

### Procedure

The questionnaire was administered via Bristol Online Survey and involved rating the subjective importance of specific characteristics sought in a partner, focusing on features related to three partner characteristics: physical attractiveness, high status, and resource acquisition abilities, and likeable personality and similar values. The survey consisted of 33 items that assessed characteristics on the three broader traits. A 7-level scale was used to assess the importance of each item, ranging from 1 (absolutely unimportant) to 7 (absolutely indispensable).

### Data Analysis

The study had an independent samples quasi-experimental design with sightedness (blind vs. sighted) and participant sex (male vs. female) as between-participant factors. All statistical analyses were carried out in R version 3.4.1. Two-way factorial ANOVAs were employed to assess the effects of participant sightedness and sex on importance ratings of the three partner characteristics. As group sizes were not balanced between the two different sightedness groups and as interactions between the factors could be expected, Type III Sum of Squares were used. Significant interactions were followed up with post hoc independent *t* tests with Bonferroni-correction to account for multiple comparisons. This adjustment was achieved by multiplying the obtained *p* value by the number of post hoc tests performed. Note that *p* values that were adjusted for multiple comparisons are reported as *p*_adj_ below.

We used a chi-square test to assess whether the proportion of individuals that believed in an indirect appearance effect differed between females and males in both vision groups. The belief in indirect appearance effects was assessed by asking participants whether they believed if their partner’s appearance influenced how others perceived them. We further carried out an exploratory analysis investigating the effect of the participants’ current relationship status on the importance placed on the three partner characteristics. In order to confirm that the questionnaire was reflective of measuring the partner characteristics physical attractiveness, status and resources, and likeable personality and similar values, we conducted a confirmatory factor analysis.

Lastly, we investigated whether blind and sighted individuals place more importance on visual, auditory, or olfactory characteristics. We compared preference ratings between the two sexes (females, males) in both vision groups (sighted, blind) for three items (looks, voice, odor) separately, using two-way factorial ANOVAs.

## Results

The 33 items assessing desired partner characteristics were first subjected to factor analysis in order to confirm that these items reflected the three following partner characteristics in line with previous studies: Physical Attractiveness, Status and Recourses, and Personality and Values. Prior to performing this analysis, suitability of the items was assessed by inspection of the correlation matrix, which revealed the presence of several coefficients with *r* > .30. Sampling was found to be adequate, as the Kaiser–Meyer–Olkin value was 0.75, which exceeded the recommended value of 0.60 (Kaiser, 1970, 1974). The factor analysis revealed the presence of eight components with eigenvalues > 1. Inspection of the screeplot indicated a break after the third component, beyond which the increase of explained variance with each component was minimal (< 1% increase with each component). Overall, this three component solution explained a total of 51.2% of the variance, supporting a structure made up of three factors: Physical Attractiveness, Status and Resources, and Personality and Values. This three factor solution was further confirmed by Horn’s (1965) parallel analysis (see Figure S1) which has been shown to be a reliable method in determining the cut off for eigenvalues in factor analysis (Velicer, Eaton, & Fava, 2000).

Item combination for the factors Physical Attractiveness and Status and Resources was based on Hasenkamp et al. (2005). Furthermore, maximum likelihood factor analysis with varimax rotation revealed the presence of a simple structure, showing high loadings (≥ 0.50) for several items across all three factors (see Table 1). The items measuring the importance of an active, vital lifestyle (0.41), sociability (0.33), same religion (NA) and same political views (NA) loaded most or were most contextually related to the factor Personality and Values. Cronbach’s alpha showed high internal consistency for all three factors (Physical Attractiveness, *α* = .91; Status and Resources, *α* = .89; Personality and Values, *α* = .78).

**Table 1:** Questionnaire items that loaded highly on the three factors

Factors	Physical attractiveness		Status/resources		Personality/values	
Questions	How important is it for a partner to…	Loadings	How important is it for a partner to…	Loadings	How important is it for a partner to…	Loadings
	Be Lean	0.87	Be of a higher social class than you	0.74	Have a reliable character	0.72
	Have a fit, firm body	0.82	Earn more than you	0.72	Have emotional stability and maturity	0.70
	Have a well-proportioned figure	0.79	Be career-oriented	0.70	Show affection and love	0.67
	Not be physically disabled	0.70	Have a distinguished social status	0.68	Be humorous	0.62
	Look good	0.70	Have a secure financial future?	0.60	Have a pleasant disposition	0.55
	Have healthy smooth skin	0.67	Be ambitious	0.58	Be active and vital	0.41
	Not show significant signs of aging	0.65	Have a school education which is similar to your school education	0.55	Have a partner who is sociable	0.33
	Have beautiful healthy hair	0.61	Be educated	0.49	Have the same political views as you	–
	Not have a language error, that is, for example, not lisp, or stutter	0.58	Be intelligent	0.39	Have the same religious background as you	–
	Be taller or shorter than you	0.50	Be diligent	0.39		

Shapiro–Wilk tests were carried out to determine whether the data met the assumptions of normality. Scores on all three factors were normally distributed within each group, with the exemption of sighted males, who showed violation of normality for the reported importance of Status and Resources (*W* = 0.92, *p*_adj_ = .028) and sighted females, who showed a violation of normality for the importance of Personality and Values (*W* = 0.92, *p*_adj_ = .034). However, Pallant (2016) indicated that violation of this assumption can be tolerated by most techniques when the sample size is larger than 30 participants. With a sample size of 94 participants, carrying out a two-way ANOVA on the data was considered appropriate. There were three data points that fell outside of 3 SDs from the mean in the factor measuring Personality and Values all of which were removed as outliers from the analysis for this factor. Levene’s test indicated that scores for Physical Attractiveness, (*F*[5, 88] < 1, *p* = .727), Status and Resource Acquisition, (*F*[5, 88] = 1.24, *p* = .298) as well as Personality and Values, (*F*[5, 85] < 1, *p* = .475), did not violate the assumption of variance homogeneity.

### Sex Differences Between Blind and Sighted Individuals

**Fig. 1:**
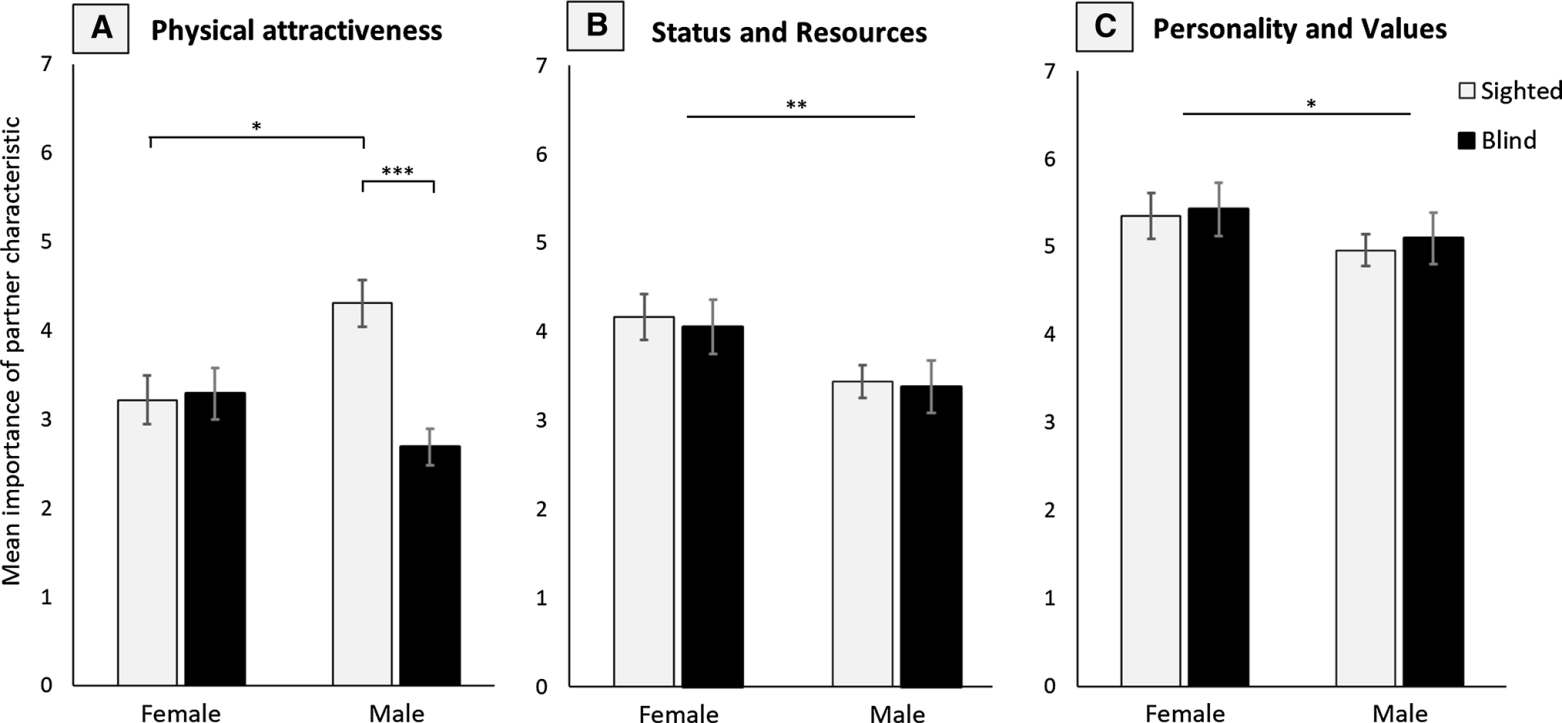
Mean scores indicating the importance of **a** physical attractiveness, **b** high socioeconomic status and resource acquisition ability, and **c** a likeable personality and similar values for sighted and blind, female and male participants. Error bars indicate standard error of the mean. *p_adj_ < .05; **p_adj_ < .01; ***p_adj_ < .001

A two-way ANOVA indicated a significant main effect of sightedness, (*F*[1, 90] = 8.76, *p* = .004, *ηp*^2^= .09), and a significant interaction between sex and sightedness for the importance of physical attractiveness, (*F*[1, 90] = 8.76, *p* = .004, *ηp*^2^= .09; see Fig. 1a). Post hoc tests revealed that the main effect was largely driven by a significant difference between blind and sighted males, (*t*[45] = 4.83, *p*_adj_ < .001). Blind and sighted females did not differ in their mean importance ratings for physical attractiveness, (*t*[42] < 0.01, *p*_adj_ > .999). In the sighted group, males reported significantly higher mean scores than females, (*t*[54] = 2.88, *p*_adj_= .023). Contrastingly, no significant difference in preference was found between males and females in the blind participant group, (*t*[33] = 1.48, *p*_adj_= .598).

Investigating group differences on the importance of status and resource acquisition ability for partner choice revealed a significant main effect of participant sex, (*F*[1, 90] = 7.31, *p* = .008, *ηp*^2^= .075). Sightedness did not show a significant main effect, (*F*[1, 90] < 1, *p* = .741) , and there was no interaction between sightedness and sex, (*F*[1, 90] < 1, *p* = .916; Fig. 1b). Overall, females gave higher ratings for the importance of high status and good resource acquisition in a partner than males, (*t*[88] = 2.8, *p* = .006).

The importance of a likeable personality and similar values in a partner significantly differed between males and females, (*F*[1, 87] = 6.06, *p* = .016, *ηp*^2^= .065, Fig. 1c); however, there was no main effect of sightedness, (*F*[1, 87] < 1, *p* = .860), and also no significant interaction between sightedness and sex, (*F*[1, 87] < 1, *p* = .810). Note that responses from three individuals were removed from this analysis as outliers (see Method). Overall, mean importance scores for personality and values were higher in female than in male participants, (*t*[89] = 2.58, *p* = .012).

### Indirect Appearance Effects

From the 94 participants, 68 believed that their partner’s appearance influences the way others perceive them (see Table 2). The proportion of participants that reported to believe in this indirect appearance effect did not differ between sighted females and sighted males, (*χ*^2^[1, *N *= 56]) = 1.02, *p* = .313); however, blind males reported to believe in the indirect appearance effect significantly less than blind females, (*χ*^2^[1, *N *= 38] = 5.4, *p* = .02).

**Table 2:** Number (proportion) of individuals who responded that their partner’s appearance influenced the way they were perceived by others

Sex	Influence	No influence
Sighted		
Female	21 (75%)	7 (25%)
Male	24 (86%)	4 (14%)
Blind		
Female	15 (79%)	4 (21%)
Male	8 (42%)	11 (58%)

**Fig. 2:**
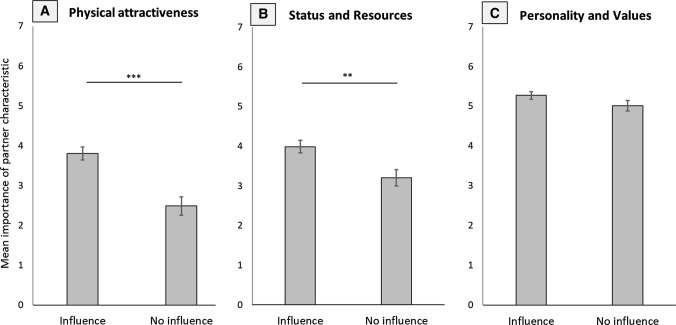
Mean scores indicating the importance of **a** physical attractiveness, **b** high socioeconomic status and resource acquisition ability, and **c** a likeable personality and similar values for individuals that reported to believe their partner’s appearance influences the way they are perceived by others and those individuals that reported to not believe in this effect. Error bars indicate standard errors of the mean. **p_adj_ < .01; ***p_adj_ < .001

Participants were split into groups based on whether they believed in the indirect influence of their partner’s appearance. Preference ratings for a partner’s physical attractiveness were significantly influenced by whether individuals believed in this indirect influence (*F*[1, 90] = 21.43, *p* < .001, *ηp*^2^= .038, Fig. 2a). There was no interaction between belief and participant sex, (*F*[1, 90] < 1, *p* = .985), or sightedness of participants, (*F*[1, 90] < 1, *p* = .584). The unbalanced grouping (see Table 2) did not allow for the assessment of a three-way interaction. Overall, individuals who believed that their partner’s appearance influences how others perceive them rated the physical attractiveness of their partner as more important, (*t*[52] = 4.71, *p* < .001).

Across groups, the importance of a partner’s high status and ability to acquire resources was modulated by whether individuals believed in this indirect influence, (*F*[1, 90] = 7.41, *p* = .008, *ηp*^2^= .076, Fig. 2b). There was no interaction of belief in this effect with participant sex, (*F*[1, 90] = 1.43, *p* = .235), or the sightedness of participants, (*F*[1, 90] < 1, *p* = .536). Overall, individuals that believed that their partner’s appearance indirectly influenced how others perceive them had higher expectations about their partner’s status and ability to acquire resources than those that did not (*t*[55] = 3.05, *p* = .004).

**Fig. 3:**
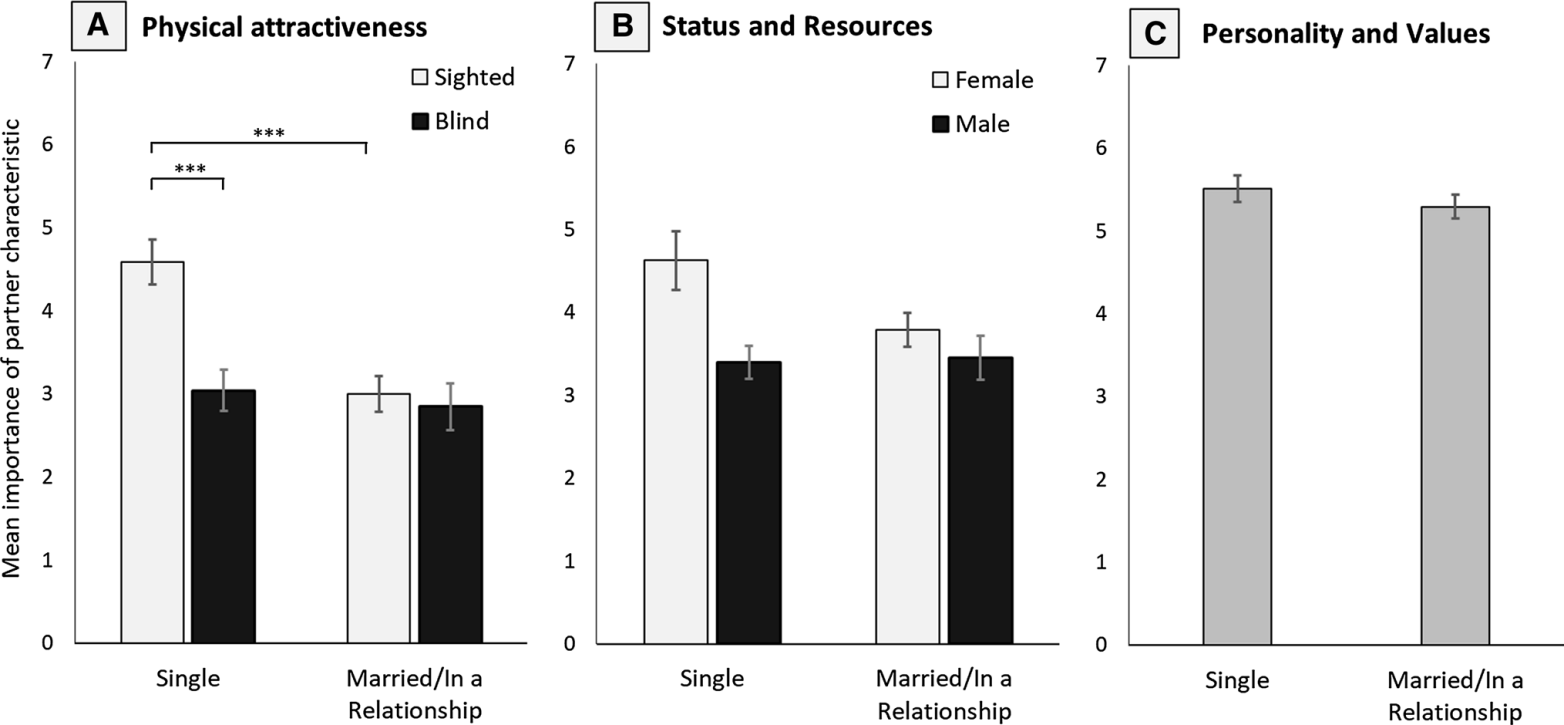
Mean scores indicating the importance of **a** physical attractiveness, **b** high socioeconomic status and resource acquisition ability, and **c** a likeable personality and similar values for individuals that reported to be single, divorced or widowed, and individuals that reported to be in a long-term relationship or married. Ratings are shown for sightedness- or sex-separated groups based on effects reported in the main text. Error bars represent standard error of the mean. ***p_adj_ < .001

The importance of personality and values was not influenced by whether individuals thought that their partner’s appearance influences how others view them or not, (*F*[1, 87] = 1.42, *p* = .237, Fig. 3b). There was no significant interaction between indirect appearance effect and participant sex, (*F*[1, 87] < 1, *p* = .361), or an individual’s sightedness, (*F*[1, 87] < 1, *p* = .569).

### Relationship Status

Participants were asked about their current relationship status at the time of the survey. A total of 38 participants reported to be single (23 male), 46 in a long-term relationship or married (18 male), 8 divorced (4 male), and 2 widowed (both male). Due to the low number of divorced and widowed individuals, and due to them not being in a current relationship, these individuals were grouped with the single individuals.

We found a significant main effect of relationship status on the importance of physical attractiveness, (*F*[1, 90] = 10.59, *p* = .002, *ηp*^2^= .016) as well as a significant interaction between relationship status and the sightedness of participants, (*F*[1, 90] = 7.19, *p* = .009, *ηp*^2^= 0.013; see Fig. 3a). Follow-up contrasts showed that sighted, single individuals placed significantly more importance on physical attractiveness compared to sighted individuals that were married or in a relationship, (*t*[51] = 4.59, *p*_adj_< .001) or single blind individuals, (*t*[46] = 4.25, *p*_adj_< .001). The importance reported by blind individuals did not differ between relationship status, (*t*[22] = 1.07, *p*_adj_> .999). There was no significant interaction between relationship status and sex of participant, (*F*(1, 90) = 1.07, *p* = .303).

There was no main effect of relationship status, (*F*[1, 90] = 2.38, *p* = .126, *ηp*^2^= .002, Fig. 3b) and no significant interaction between relationship status and sex for the importance of status and resources, although this was close to significance, (*F*[1, 90] = 3.16, *p* = .079, *ηp*^2^= .003), with single females reporting the highest importance of this partner characteristic. Single males, married males, and married females reported lower importance scores. There was also no interaction between sightedness and relationship status on the importance of status and resources (*F*[1, 90] < 1, *p* = .808).

We found no main effect of relationship status on the importance of a likeable personality and similar values, (*F*[1, 87] < 1, *p* = .670, Fig. 3c), and no significant interaction between sightedness and relationship status, (*F*[1, 87] < 1, *p* = .471), or sex and relationship status (*F*[1, 87] < 1, *p* = .853). Overall, single, divorced, and widowed individuals did not differ from participants that were married or in long-term relationships.

### Importance of Other Senses in Blind and Sighted Individuals

**Fig. 4:**
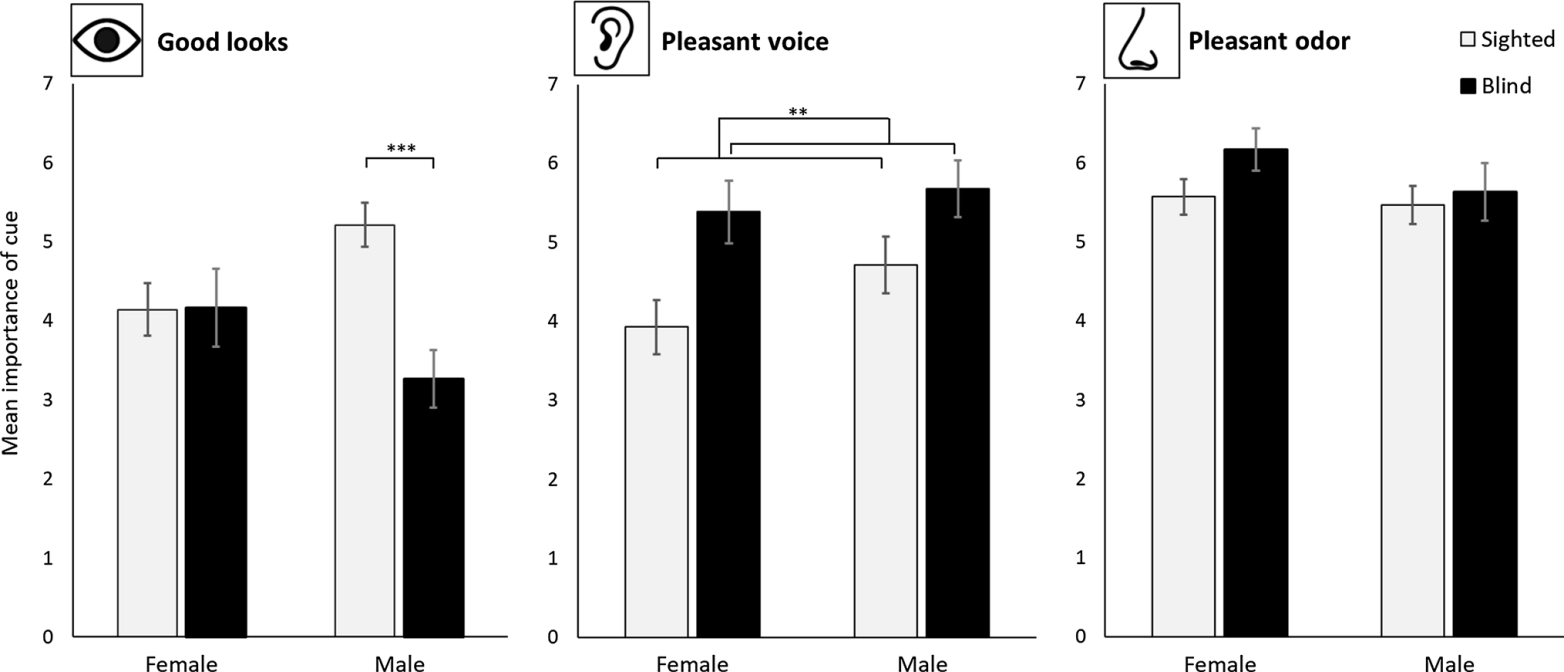
Mean importance scores for three different partner characteristics, referring to the use of three different senses: vision (good looks), hearing (pleasant voice), and smell (pleasant odor). Scores are presented for sighted and blind females and males separately. Error bars indicate standard error of the mean. **p_adj_ < .01; ***p_adj_ < .001

In order to assess the importance of the different senses for partner preference between sighted and blind men and women, preference ratings were compared between three single items: good looks, a pleasant voice, and a pleasant odor (Fig. 4).

There was a significant interaction between sex and sightedness for the importance of good looks, (*F*[1, 89] = 7.37, *p* = .008, *ηp*^2^= .012). Post hoc tests revealed that this interaction was largely driven by differences in males. Here, good looks were considered more important by sighted males than blind males (*W* = 434.5, *p*_adj_ < .001). There was no difference between sighted and blind females (*W* = 251, *p*_adj_ > .999). Following adjustment for multiple comparisons, we found no significant sex differences for the sighted (*W* = 253, *p*_adj_ = .083) and the blind (*W* = 213.5, *p*_adj_ = .785) groups.

There was a main effect of sightedness on the importance of a pleasant voice, (*F*[1, 89] = 10.53, *p* = .002, *ηp*^2^= .013), with blind individuals indicating higher importance for this trait than sighted individuals. There was no difference between males and females, (*F*[1, 89] = 2.08, *p* = .152), and no interaction between sex and sightedness (*F*[1, 89] < 1, *p* = .514).

A pleasant odor was considered equally important by all groups, and there was no effect of sex, (*F*[1, 89] = 1.37, *p* = .244), or sightedness, (*F*[1, 89] = 1.94, *p* = .168), on the importance ratings of this trait. There was also no interaction between sex and sightedness, (*F*[1, 89] < 1, *p* = .437).

Lastly, we compared importance ratings between the three different traits for sighted and blind participants separately. As scores violated the assumption of normality in several groups (as assessed by Shapiro–Wilk with *p* < .05), six Bonferroni-corrected Wilcoxon-signed rank tests were conducted to investigate the contrasts between the single items and between sighted and non-sighted individuals. Importance ratings for all three characteristics are displayed in Fig. 4. Results indicated that, in sighted participants, a pleasant odor was rated as most important compared to either good looks (*V* = 378, *p*_adj_ = .003) or a pleasant voice (*V* = 631, *p*_adj_ < .001). There was no significant difference between the importance ratings of good looks and a pleasant voice (*V* = 442, *p*_adj_ = .993). Blind participants also considered a pleasant odor significantly more important than good looks (*V* = 496, *p*_adj_ < .001), but not more important than a pleasant voice (*V* = 162.5, *p*_adj_ = 1). A pleasant voice was also rated as significantly more important than good looks (*V* = 451, *p*_adj_ < .001).

## Discussion

Our study investigated sex differences in mate preference between sighted and blind individuals for the importance of three partner characteristics: Physical Attractiveness, Status and Resource Acquisition Ability, and Personality and Values. We furthermore assessed the importance of different sensory cues for partner choice in sighted and blind individuals.

Our results showed that physical attractiveness was more important for sighted than for blind men, while sighted and blind women did not differ in the importance they placed on this partner characteristic. This is consistent with previous findings (Hasenkamp et al., 2005) showing a decrease in importance ratings for this factor in blind men relative to blind women. It is also worth noting that several of the characteristics for which preferences were assessed can be perceived by senses other than vision (e.g., a person’s height, healthy smooth skin, or lack of language error). The lesser importance of attractiveness in blind men suggests that the preference for certain partner characteristics is influenced by individual sensory experiences. However, this does not undermine the desire of males to obtain a fertile and healthy partner. That is, as a lack of vision does not allow the direct assessment of female fertility and health through visual attractiveness, these traits might be assessed in other ways, such as through auditory or olfactory cues, or indirectly through the report of sighted friends. In line with a compensation in sensory cue use, we found that the importance of an auditory cue, a pleasant voice, was higher in blind individuals.

More surprisingly, however, was our finding that blind and sighted females did not differ in their reported desirability of a physically attractive mate. A potential explanation for this lies in the indirect appearance effects of physical attractiveness. That is, whether an individual believes their partner’s appearance influences how others perceive them might affect their desire to be with a physically attractive partner. As having a physically attractive partner increases an individual’s own attractiveness (Little et al., 2011), this might yield a benefit by appearing more attractive to others and might therefore explain why both blind and sighted women considered physical attractiveness equally important. Notably, while the majority of blind females reported that their partner’s appearance has an influence on how others perceive them, the majority of blind males did not believe in this indirect effect. This suggests that the importance blind females place on other people’s perceptions may be linked to their own level of desirability as romantic partners. This increased focus on own appearance might be especially important as visually impaired and blind individuals are often less likely to be viewed as potential dating or marriage partners (Fichten, Goodrick, Amsel, & McKenzie, 1991) and the likelihood of being single becomes greater in relation to the severity of a physical disability (DeLoach, 1994; Taleporos & McCabe, 2003). Disabled people have furthermore been reported to internalize the negative reactions and attitudes that people have toward them, leading to poorer body image (Taleporos & McCabe, 2002). Therefore, blind women may be more inclined to believe that their choice of partner influences how others view them.

This finding further questions what females gain from a physically attractive male partner—not least because the literature on direct benefits (e.g., investment) of a physically attractive male partner is inconsistent (but see Little, Jones, Feinberg, & Perrett, 2014). The interaction we reported here would suggest that physical attractiveness might be conceptualized differently in males and females. That is, when men judge physical attractiveness, this seems to reflect a focus on cues that are linked to fertility, and which can be assessed more easily through vision. For women, on the other hand, physical attractiveness might be more complex, involving greater focus on behavioral and investment cues. This idea is also supported by findings from a recent study on the perception of facial qualities, which used multivariate regression to generate images of faces that varied along three different facial composites: attractiveness, dominance and trustworthiness (Jones, 2018). Participants were asked to rate the attractiveness of male and female faces that have been generated with high and low levels of attractiveness, while controlling for trustworthiness and dominance. As the latter two characteristics often co-vary with attractiveness, this approach offers a way to disentangle the evoked perception of facial attractiveness. The results indicated that female faces were largely judged based on attractiveness alone, independently of trustworthiness and dominance. However, when male faces were judged, attractiveness seemed to be largely influenced by dominance and trustworthiness (Jones, 2018). These findings would support our idea that attractiveness might be interpreted differently by males and females, such that in males, it serves as an indicator for fertility, while in females it provides a proxy for social dominance (an indicator of social status) and trustworthiness (an indicator of similar personality and values).

Moreover, the current study finds blind participants to not value a partner’s status and resource acquisition ability any differently than their sighted counterparts. This contrasts with Hasenkamp et al.’s (2005) findings that showed lower importance of a partner’s status and resource acquisition ability in blind compared to sighted individuals. We found that overall, both sighted and blind women demonstrated a stronger preference for a partner with good financial prospects than men did. This is not surprising given that status and resource acquisition in our study were measured by partner characteristics that could be assessed non-visually, such as being-career-oriented, ambitious, or having a higher income (see Table 1 for a list of items). Therefore, the findings would support the evolutionary approach that sex-specific mate preferences have evolved as a functional adaptation to producing and raising high-quality offspring.

Similar to status and resource acquisition, preferences for a similar personality and values were not influenced by sightedness and were higher in females than in males. We did not find a difference between sighted and blind participants, as predicted based on findings by Pinquart and Pfeiffer (2012). However, this might be explained by Pinquart and Pfeiffer focusing specifically on emotional maturity, which was described as a partner’s dependability, faithfulness, honesty, and kindness. The characteristics assessed in the present study more broadly concerned the preference for a likeable personality and similar values (see Table 1), including further factors that relate to people’s lifestyle choices. While emotional maturity might be more important for visually impaired and blind individuals, sighted individuals potentially place more importance on other personality characteristics. This also highlights that inter-individual variability in the preference for specific traits might be higher, while a lower sex-specific variability in the preference for more general characteristics (i.e., physical attractiveness, status and resources) would underlie more universal mechanisms that can be explained within the evolutionary framework. Future research could establish a more differentiated picture of the importance of different personality traits and the different roles that self-other similarity and social desirability play for preferences in a partner’s personality.

One limitation of this study, which is also mirrored in the majority of literature on mate preferences, is that these findings are limited to heterosexual mate choice. With an increasing recognition and openness toward a more fine-grained spectrum of sexual identity and sexual orientation (e.g., Thompson & Morgan, 2008; van Anders, 2015), a better understanding of non-heterosexual mate choice would allow deeper insights into the emergence of mate preferences, while acknowledging the existing diversity of sexuality across all populations. Furthermore, blind participants are a difficult to recruit sample and we also note that future studies would benefit from larger sample sizes alongside more diverse samples in terms of sexuality and culture. Additionally, our research is limited to the questions which were largely developed for sighted participants, and future insight could benefit from additional data gained through structured qualitative interviews which could enable better expression of less typical sensory experiences.

Social factors that we found influenced partner preferences were relationship status and whether the individual thinks their partner’s appearance influences how others perceive them. In sighted, but not in blind individuals, relationship status influenced the desirability of physical attractiveness, and there was a trend for the importance of status and resources. Here, single participants placed more importance on these characteristics than those participants that were in a relationship. However, relationship status did not alter preferences for physical features and resources in blind individuals. These findings are consistent with results from previous studies reporting that partnered individuals place less value on physical attractiveness than single individuals and may be related to partnered individuals’ desire to protect their current relationship (Karremans et al., 2011; Miller & Maner, 2010; Ritter & Karremans, 2010).

Partner characteristics differ in terms of their accessibility through different senses. Given that vision is absent in blind individuals we might expect that the assessment of partner characteristics based on this modality would differ between blind and sighted individuals. At the same time, due to an increased importance of the other senses to convey sensory information, we would also expect that the value of cues from the other modalities would be higher in the blind. We found that sighted participants reported higher importance for good looks whereas blind participants reported an increased importance of a pleasant voice. A pleasant odor was rated as most important in both groups. This is in line with Sorokowska et al.’s (2018) study showing that blind individuals, compared to sighted individuals, place more importance on auditory cues while considering odor qualities as equally important.

Sex differences were detected only for visual cues. As expected, visual cues were most important for sighted males, perhaps because they provide more direct information about a partner’s age and fertility than auditory information arising from voice (Buss, 2006; Havlíček et al., 2008; Moyse, 2014). Visual cues were less important for blind males, probably because they could not be directly assessed and therefore do not offer useful information. In line with the finding that blind women place similar importance on a partner’s physical attractiveness as sighted women, we also found that visual cues were equally important for sighted females and blind females. This would support our previous suggestion that indirect appearance effects might enhance the desire of females to obtain a physically attractive partner. On the other hand, it is also possible that, for females, visual physical attractiveness does not provide much additional information about a mate’s quality that cannot be conveyed through other senses. It is not known whether blind individuals would glean further information from a potential partner’s voice about, for example, age. In a recent study, blind people have been shown to not perform better than sighted people in estimating an individual’s height based on their voice (Pisanski, Oleszkiewicz, & Sorokowska, 2016). Olfactory cues, on the other hand, may have more general and far ranging importance in sighted and blind men and women alike which would explain the relatively high importance placed on odor by all groups. Chemical cues have repeatedly been shown to play an important role in human social communication, giving indications about an individual’s health (Olsson et al., 2014), genetic compatibility (Milinski, Croy, Hummel, & Boehm, 2013; Sorokowska, Pietrowski, et al., 2018), sexual arousal (Gelstein et al., 2011) as well as non-sexual functions (Schaal, 2010; Semin & de Groot, 2013).

### Conclusion

In the present study, we assessed the role that vision plays for the development of sex differences in partner preferences. We replicated well-documented findings of sex-specific mate preferences in sighted men and women. While men considered physical attractiveness relatively more important in a potential partner, women assigned more importance to wealth and a high social status. While blind men placed less importance on physical attractiveness, blind women did not differ from sighted women. This suggests that a partner’s physical attractiveness might bear an indirect advantage to women by increasing their desirability, while sighted men assess physical attractiveness as a direct indicator for fertility and health. Overall, participants who reported that their partner’s appearance influences how they are perceived by others were more concerned about their partner’s physical appearance and status. Furthermore, relationship status had an influence on how important participants rated a partner’s physical attractiveness, with single, divorced, or widowed individuals reporting a higher importance compared to individuals that were married or in a long-term relationship. The relatively higher importance that women place on socio-economic status and on a likable personality and values in men was not influenced by sightedness or relationship status. In assessing the importance of different sensory cues for partner choice, we found that blind individuals considered auditory cues more important, while sighted males considered visual cues more important. A good odor was generally rated as more important than other cues in all groups, emphasizing the importance of odor in human social communication. Overall, our findings shed light onto the role that visual experience plays for the emergence of sex differences in mate preferences, suggesting that evolutionarily adaptive drivers of mate choice are influenced by individual sensory experiences.

## Supplementary Information

Supplementary file1 (DOCX 56 kb)
