# Supplementary material for: The Role of Vision in the Emergence of Mate Preferences
Source: Arch Sex Behav. 2021 Apr 13;50(8):3785–97. doi: 10.1007/s10508-020-01901-w (PMC8604830; doi:10.1007/s10508-020-01901-w)
Supplement: Supplementary file 1 — Supplementary file1 (DOCX 56 kb) [file 10508_2020_1901_MOESM1_ESM.docx]

**Supplementary Material**

**S1. Factor Analysis – Scree Plot**


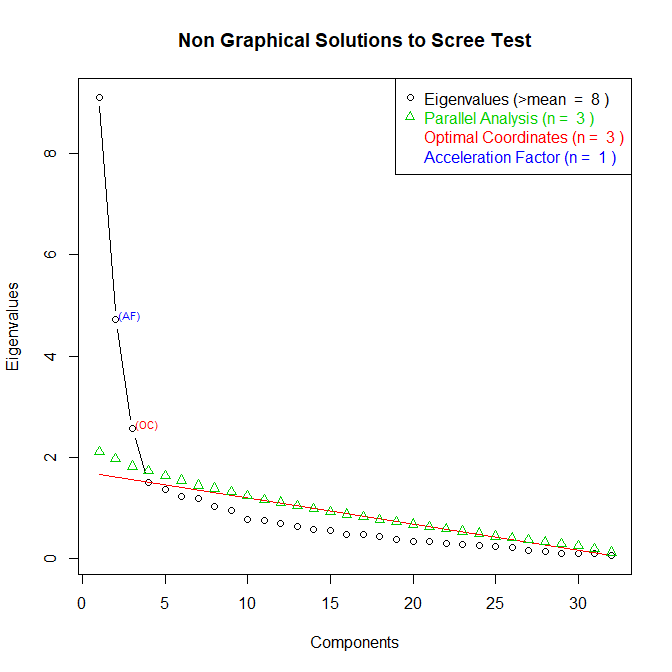


*Figure S1:* Scree plot indicating the eigenvalues for factor analysis of the 33 items assessing different dimensions of mate preference.

**S2. Differences between congenitally and late blind individuals**

In order to assess whether the sex differences in mate preferences observed between sighted and blind individuals were influenced by the onset of blindness, we compared the sub-group of congenitally blind individuals with those individuals that went blind after birth. Blindness onset in the latter group ranged from 2 years to 60 years of age. Thereby, the duration of blindness in the late-blind ranged from 4 years to 43 years. As the group of blind individuals was split up into smaller sub-groups (congenitally blind: 10 males, 10 females; late blind: 9 males, 9 females), sample sizes were too small to detect significant effects. However, average ratings should illustrate potential differences between congenitally or late blind individuals.

Mean importance ratings for a partner’s physical attractiveness are depicted for congenitally blind, late blind, and sighted individuals in figure S2. They showed that importance ratings were similar across females in all three vision groups, while they were decreased in both blind male groups (*Δ_cong_* = -0.5; *Δ_late_* = -0.6) and enhanced in sighted males (*Δ* = 1.1). However, using independent t-tests these differences did not reach significance (congenitally blind: *t*(17) = 0.88, *p* = .392, late blind: *t*(12) = 1.42, *p* = .183).

*Figure S2*: Mean importance ratings for a partner’s physical attractiveness for congenitally blind and late blind individuals plotted separately. Scores of sighted individual are plotted as a reference. Error bars indicate standard error of the mean. Gray dashed line indicates mean score of all female groups.

Mean importance ratings for a partner’s status and resources in congenitally blind, late blind, and sighted individuals are shown in figure S3. As suggested by the main analysis, the average ratings were higher for females than males in all groups (*Δ_cong_* = 0.6; *Δ_late_* = 0.8; *Δ_sighted_* = 0.7). However, also in this no significant sex-differences were found in the two blind sub-groups (congenitally blind: *t*(18) = 0.9, *p* = .38, late blind: *t*(15) = 1.62, *p* = .127).

*Figure S3*: Mean importance ratings for a partner’s status and resources for all sightedness groups plotted separately. Scores for sighted individual are plotted as a reference. Error bars indicate standard error of the mean. Gray dashed line indicates mean score of all female groups.

Figure S4 shows mean importance ratings for a partner’s similar personality and values in congenitally blind, late blind, and sighted individuals. The average ratings were higher for females than males in all groups (*Δ_cong_* = 0.5; *Δ_late_* = 0.1; *Δ_sighted_* = 0.4). Similar to above, this sub-group comparison did not reach significance for either congenitally blind (*t*(18) = 1.41, *p* = .175) or late blind (*t*(13) = 0.17, *p* = .87) individuals.

*Figure S4*: Mean importance ratings for similar personality and values in a partner for all sightedness groups plotted separately. Scores for sighted individual are plotted as a reference. Error bars indicate standard error of the mean. Gray dashed line indicates mean score of all female groups.

Overall, we did not find significant differences between males and females for congenitally blind or late blind individuals separately, which is likely due to reducing the sample size for each group. However, average importance scores for the different partner characteristics mirror the findings of the main analysis, suggesting that both congenitally blind and late blind individuals rated partner characteristics more similarly to each other, compared to sighted individuals. More specifically, physical attractiveness was rated as less important in males who were both blind from birth, or lost their vision later in life, compared to females in the respective blindness-onset groups. Sighted individuals however, showed the opposite effect, with sighted males placing higher importance on physical attractiveness than sighted women. Taken together, this might suggest that the male preference for a physically attractive partner is shaped more directly by visually perceivable cues rather than by visual memory of physical attractiveness.

**S3. Material - Questionnaire**

**Questionnaire Survey**

What is your sex? *Required*

Please select exactly one answer.

 Male

 Female

Please assess the importance of the following characteristics in a potential long-term partner on a scale from 1 (absolutely unimportant) to 7 (absolutely indispensable).

Please do not select more than one answer per row.

Please select at least 1 answer(s).

|  | 1 Absolutely Unimportant | 2 | 3 | 4 | 5 | 6 | 7 Absolutely Indispensable |
| --- | --- | --- | --- | --- | --- | --- | --- |
| How important is it for you that a partner has long hair? |  |  |  |  |  |  |  |

What is your age? *Required*

Where do you live? *Required*

What age should your partner be compared to you? *Required*

 Older

 Younger

 Same age

 No preference

If your ideal partner is older than you, how much older should they be?

If your ideal partner is younger than you, how much younger should they be?

Do you believe your partner’s appearance influences how other people perceive you? *Required*

 Yes

 No

Are you…? *Required*

 Single

 Married/in a marriage-like partnership/long-term relationship

 Separated

 Divorced

 Widowed

If you’re married, or in a long-term relationship, is your partner…?

 Able to see

 Visually impaired

 Blind from birth

 Went blind later on

If you're married, or in a long-term relationship, who is the main earner in your household?

 Me

 My partner

 We both earn the same amount

 Neither

 Other

If you selected Other, please specify:

What is the highest level of education you have completed? *Required*

 GCSEs/CSEs/O-Levels

 A Levels

 Undergraduate Degree/Bachelor’s Degree

 Masters Degree/Postgraduate Degree

 PhD

What is your current occupation? *Required*

 Employed

 In Education

 Housewife / Housekeeper

 Retired

 Unemployed

If you are working, do you work...?

 Part time

 Full time

If you are working, what sector do you work in?

What is your level of visual disability? *Required*

 Fully sighted

 Vision corrected by lenses

 Low vision or partially sighted

 Registered blind or legally blind

**Questions Page 2**

At what age did you develop blindness or visual impairment? *Required*

What is the cause of your visual impairment or blindness? *Required*

 Congenital Retinopathy

 Optic Neuropathy

 Diabetic Retinopathy

 Macular Degeneration

 Retinitis pigmentosa

 Cataract

 Glaucoma

 Other

If you selected Other, please specify:

How important is it for you that your partner possesses some form of sight (not 100% blind)?*Required*

Please don't select more than 1 answer(s) per row.

Please select at least 1 answer(s).

|  | 1 Absolutely Unimportant | 2 | 3 | 4 | 5 | 6 | 7 Absolutely Indispensable. |
| --- | --- | --- | --- | --- | --- | --- | --- |
| Please select |  |  |  |  |  |  |  |

For those who became developed a visual impairment/blindness later, do you still have a visual image when you think of someone who is attractive? *Required*

 Yes

 No

 Not applicable

**Questions Page 3**

Please assess the importance of the following characteristics in a potential long-term partner on a scale from 1 (absolutely unimportant) to 7 (absolutely indispensable).

|  | *Required* | | | | | | |
| --- | --- | --- | --- | --- | --- | --- | --- |
|  | 1 Absolutely Unimportant | 2 | 3 | 4 | 5 | 6 | 7 Absolutely Indispensable |
| How important is it for a partner to cook well and be a good housekeeper / housekeeper? |  |  |  |  |  |  |  |
| How important is it for a partner to have a healthy smooth skin? |  |  |  |  |  |  |  |
| How important is it for a partner to have a pleasant disposition? |  |  |  |  |  |  |  |
| How important is it for you to have a partner who is sociable? |  |  |  |  |  |  |  |
| How important is it for a partner to have a school education which is similar to your school education? |  |  |  |  |  |  |  |
| How important is it for you that a partner has beautiful healthy hair? |  |  |  |  |  |  |  |
| How important is it for a partner to be elegant? |  |  |  |  |  |  |  |
| How important is it for your partner to be educated? |  |  |  |  |  |  |  |
| How important is it for a partner to have a fit, firm body? |  |  |  |  |  |  |  |
| How important is it for a partner to have a secure financial future? |  |  |  |  |  |  |  |
| How important is it for you that a partner has little sexual experience? |  |  |  |  |  |  |  |
| How important is it for a partner to be active and vital? |  |  |  |  |  |  |  |
| How important is it for a partner to have a reliable character? |  |  |  |  |  |  |  |
| How important is it for a partner to have emotional stability and maturity? |  |  |  |  |  |  |  |
| How important is it for a partner to have a male figure (wide shoulders and narrow hips), or that a female partner has a narrow figure and is well-proportioned? |  |  |  |  |  |  |  |
| How important is it for you that a partner would like a home and children? |  |  |  |  |  |  |  |
| How important is it for you to have a partner of a higher social class than you? |  |  |  |  |  |  |  |
| How important is it for a partner to look good? |  |  |  |  |  |  |  |
| How important is it for you that a partner has the same religious background as you? |  |  |  |  |  |  |  |
| How important is it for you that a partner is ambitious? |  |  |  |  |  |  |  |
| How important is it for a partner to have a pleasant voice? |  |  |  |  |  |  |  |
| How important is it for a partner to be humorous? |  |  |  |  |  |  |  |
| How important is the affection and love of a partner? |  |  |  |  |  |  |  |
| How important is it for you that a partner is healthy, so does not suffer from a chronic illness? |  |  |  |  |  |  |  |
| How important is it for a partner to have a distinguished social status? |  |  |  |  |  |  |  |
| How important is it for a partner to have a pleasant body odour? |  |  |  |  |  |  |  |
| How important is it for a partner to be intelligent? |  |  |  |  |  |  |  |
| How important is it for a partner to have the same political views as you? |  |  |  |  |  |  |  |
| How important is it for a partner to be diligent? |  |  |  |  |  |  |  |
| How important is it for you that a partner does not have a language error, that is, for example, not lisp, or stutter? |  |  |  |  |  |  |  |
| How important is it for you that a partner is career-oriented? |  |  |  |  |  |  |  |
| How important is it for you that a partner is taller than you, or that a partner is shorter than you? |  |  |  |  |  |  |  |
| How important is it for you that a partner is lean? |  |  |  |  |  |  |  |
| How important is it for you that a partner is not physically disabled? |  |  |  |  |  |  |  |
| How important is it for a partner to earn more than you? |  |  |  |  |  |  |  |
| How important is it for you that your partner does not show significant signs of aging? |  |  |  |  |  |  |  |
| How important is it for a partner to be cared for? |  |  |  |  |  |  |  |
